# Coping with Spatial Heterogeneity and Temporal Variability in Resources and Risks: Adaptive Movement Behaviour by a Large Grazing Herbivore

**DOI:** 10.1371/journal.pone.0118461

**Published:** 2015-02-26

**Authors:** Jodie Martin, Simon Benhamou, K. Yoganand, Norman Owen-Smith

**Affiliations:** 1 Centre for African Ecology, School of Animal, Plant and Environmental Sciences, University of the Witwatersrand, Johannesburg, South Africa; 2 Centre d’Ecologie Fonctionnelle et Evolutive, CNRS UMR5175, Montpellier, France; Institut Pluridisciplinaire Hubert Curien, FRANCE

## Abstract

Movement is a key mean for mobile species to cope with heterogeneous environments. While in herbivorous mammals large-scale migration has been widely investigated, fine-scale movement responses to local variations in resources and predation risk remain much less studied, especially in savannah environments. We developed a novel approach based on complementary movement metrics (residence time, frequency of visits and regularity of visits) to relate movement patterns of a savannah grazer, the blue wildebeest *Connochaetes taurinus*, to fine-scale variations in food availability, predation risk and water availability in the Kruger National Park, South Africa. Wildebeests spent more time in grazing lawns where the grass is of higher quality but shorter than in seep zones, where the grass is of lower quality but more abundant. Although the daily distances moved were longer during the wet season compared to the dry season, the daily net displacement was lower, and the residence time higher, indicating a more frequent occurrence of area-concentred searching. In contrast, during the late dry season the foraging sessions were more fragmented and wildebeests moved more frequently between foraging areas. Surprisingly, predation risk appeared to be the second factor, after water availability, influencing movement during the dry season, when resources are limiting and thus expected to influence movement more. Our approach, using complementary analyses of different movement metrics, provided an integrated view of changes in individual movement with varying environmental conditions and predation risk. It makes it possible to highlight the adaptive behavioral decisions made by wildebeest to cope with unpredictable environmental variations and provides insights for population conservation.

## Introduction

In spatially and temporally heterogeneous environments, movement is a key mechanism allowing animals to cope with highly dynamic resource productivity. Many large herbivores are known to migrate over large spatial scales to accommodate seasonal variation in forage production and retention [1–5]. Much less information is available on how animals move over finer scales in response to local temporal and spatial variation in resources and risks, especially in savannah environments.

Scale is acknowledged as a key concept when studying animal movements [6]. For moose (*Alces alces*) which inhabit northern temperate environments, Van Moorter et al. [7] developed an integrative approach to relate the scale of movement to the scale of resource variability in space and time. They showed that scale of movement was related to scale of environmental changes. Large scale waves of change triggered migration and fast ripples of change in resource availability at small spatial scales induced longer daily movement. When the environment was productive and the fine-scale changes in resource availability were fast, moose moved more frequently over shorter time lags. However, in arid or semi-arid savannah environments, production pulses, mainly governed by rainfall, are irregular and thus unpredictable in time. Furthermore, herbivores may maintain local grass swards in a short-cropped state of high nutritional value, referred to as “grazing lawns” [8], [9] that are even less predictable in time. Although spatially predictable, available forage biomass depends on the interplay between grazing and grass re-growth, with the latter dependent on rainfall and temperature conditions. Hence these grazing lawns exhibit pulsed dynamics between resource production and consumption by herbivores [9]. In these environments, large herbivores therefore experience strong but somewhat erratic seasonal variations in resource abundance and quality, which are inversely correlated [10].

Herbivore movements are additionally influenced by predation risk and, in arid or semi-arid environments, by water availability, which both vary in space and time as well. Predators like lions (*Panthera leo*) may concentrate near waterholes because of herbivore aggregations in the vicinity [11]. Ambush predators also preferentially hunt in proximity to cover [12], and more successfully at night than during the day.

Time spent in particular areas and frequency of return visits provide complementary measures of how individuals respond to spatially and temporally heterogeneous resources and risks. GPS technology facilitates high resolution tracking of animal movements over extended time periods. New methods have been proposed to identify and distinguish areas of prolonged residence from areas frequently visited [13–15]. Grazing patterns by large herbivores are of particular interest because they take place within a vegetation context that is variable over time. Selective preferences for particular types of food resources can be expressed through (1) staying longer in areas with higher profitability and/or (2) frequently returning to such areas [16], [17] depending on the balance between forage accumulation and regeneration. However, animals should not remain very long in specific places and revisit them less regularly to reduce their predictability for predators and thus encounter rates [18].

In this paper, we aimed at relating fine-scale environmental variations and herbivore movements to their functional implications for resource acquisition and predator avoidance using an integrative approach. We investigated how a grazer, the blue wildebeest (*Connochaetes taurinus*) tracks and exploits fine-scale variation in food resources, taking into account predation risk and water availability. Blue wildebeests are water-dependant grazers that preferentially concentrate in grazing lawn grasslands, switching to grasslands retaining adequate biomass during the early dry season [19].

We investigated: (1) what influences the duration of stay in a particular place, (2) what influences recursions to previously visited places, and (3) what influences the regularity of visits to particular places. For any place, we estimated the residence time, as well as the frequency and regularity of return visits within seasons, and assessed the influences of external drivers: food resources, predation risk and proximity to water on these movement features, and particularly their relative importance. From optimal foraging principles [20] and concepts developed by Van Moorter et al. [7], we expect that: (a) during the wet season, when food resources are abundant and spatially predictable, recursions to previously visited areas should be frequent as wildebeest graze preferentially on short grass, but with a low residence time to efficiently exploit resources of highest quality; in contrast, during the dry season, when the availability of green grass is less predictable and abundant, we expect the foraging behavior to change (with respect to wet season), with re-visits to previously exploited places less frequent because the grass is not re-growing and a longer giving-up time because the overall productivity in the landscape is low. We also expect that (b) wildebeest should leave patches of high-quality food resources (grazing lawns) sooner compared to patches with lower quality but higher quantity of food resources because they are less rapidly depleted. However, the return rate should be higher in grazing lawns to closely track re-growing grass. We also expect that (c) during the wet season, movement should be influenced more by predation because resources are abundant and more widely distributed whereas during the dry season resources should be the main influence. Finally, (d) water should be one of the main determinants of movement during the dry season but because of the higher predation risk at close proximity to water, we expect a low residence time but high frequency of visits at water by water-dependent wildebeest.

## Material and Methods

### Study area and species

Our study took place from April 2009 to April 2011 in the west-central part of the Kruger National Park (Orpen Gate region), and the adjoining Manyaleti Game Reserve and Timbavati Private Nature Reserve. The study area extended between longitudes-24.30°S and-24.75°S and between latitudes 31.35°E and 31.55°E, and covered approximately 500 km². The landscape incorporated an intrusion of gabbro substrate within the surrounding geological matrix of granitic-gneiss. Gabbro substrates generate clay-rich soils of comparatively high fertility, while granitic soils are sandy and less fertile. A feature of the gabbro upland was the prevalence of open areas with short grass resulting from concentrated grazing pressure, hereafter referred to as “grazing lawns”, within a matrix of medium/tall grass. The granitic region included seep-zone grasslands (hereafter “seep zones”) in mid-slope regions that retained green grass for longer period into the dry season. Although together, grazing lawns and seep zones covered only 10% of the study area, these habitats were the most favoured by wildebeest with 63–78% of the collared herds being located in these habitats during grazing times of day [19]. The surrounding habitat mosaic was typified by medium/tall grass areas and greater tree canopy cover. Approximately 800 wildebeests inhabited the study area, along with *c*.*a*. 7000 impalas (*Aepyceros melampus*), *c*.*a*. 900 plain zebras (*Equus burchelli*) and *c*.*a*. 600 African buffalos (*Syncerus caffer*) as major grazers (estimated from aerial counts conducted through 1980s until 1995). Three lion prides’ range encompassed the study area, representing an estimated number of 33 individuals (unpublished data).

Rainfall mainly occurs during summer with 75–80% of the total annual rainfall occurring from November to March [21]. During this warm, wet season, the association between rainfall and grazing activities maintain the grass in a growing stage of development and thus of good quality and quantity [22]. Temperature and food availability and quality then decline progressively during the dry season. Late in the dry season, forage is scarce and contains much less nutrients. In the study area, the rainfall averages 570mm per year (unpublished data from the ranger station Kingfisherspruit, from 1960). During our study period, the environmental conditions were representative of the prevailing conditions in the study area with a total rainfall of 526 mm from May 2009 to April 2010 and 618mm from May 2010 to April 2011.

### Ethics statement

The GPS data base that we used was provided by a research project previously registered with South African National Parks (see [19]). Animal capture to place the GPS/GSM collars for that study had been conducted by their scientific staff following their ethical guidelines and did not involve endangered or protected species. The weight of the GPS/GSM collars was 1.08 kg. This is only 0.5% to 1% of the body weight of an adult female wildebeest that we deployed the collars on. It was thus assumed that the collars did not interfere with normal behaviour nor alter movements of animals and herds. The collars were removed at the end of the study period.

### GPS data

In total, nine female wildebeest in nine independent herds (S1 Table), each consisting of 12–35 animals and representing 40% of the herds in the study area (estimated from relative sightings of collared versus unmarked herds; [19]) were used for this study. The collars provided hourly GPS relocations from April 2009 to April 2011 (S2 Table for details on each herd).

No activity data were recorded, but were derived from movement rates. As wildebeest are mainly grazers, they tend to move when feeding, contrary to browsers [23]. In average, they were observed to move about 200 m per hour while foraging (Yoganand unpubl.). Resting bouts were defined as periods involving movements less than 50 m between successive hourly GPS locations, corresponding to possible false movements generated by the GPS errors, which averaged 10m (based on field assessments with collar set at fixed location). This averaged value is measured as the dispersion of locations around their barycentre so that 95% of the locations are within 25–30m. However, for identifying resting behavior, the error to be considered is the one measured between successive locations (i.e. on differences in location), which is higher than the error measured at fixed location. Using a threshold value of 50 m thus is a reliable and conservative mean to distinguish resting and moving without activity sensors and remove all resting locations. Foraging bouts were defined as periods involving movements of 50–250 m and travelling bouts as periods involving movements larger than 250 m. We cannot exclude that some tracked segments that are mixing ranging and resting could have been erroneously rated “feeding” but we are confident that this should occur scarcely because the main behaviors (resting, feeding, ranging) were usually performed for a while. For the analyses, we did not consider intervals between GPS relocations that were longer than 1 hour.

### Habitat and environmental covariates

Three seasons were distinguished based on rainfall patterns and grass growth responses, plus a transitional month. Roughly, the wet seasons extended from December to April, the early dry seasons from May to July, and the late dry seasons from August to October, with the transitional months being November. During these transitional months, rainfall events started but were not evenly distributed throughout the study area. They thus strongly varied amongst herds, which made it difficult to obtain reliable and general results for these months. We provide the exact dates in S3 Table. Given that GPS data extended over two years, each season is replicated twice.

Habitat types were mapped using a SPOT5 satellite image (CNES, Toulouse, France) dated May 2006, categorized as grazing lawns, seep zones, medium/tall grass and wooded areas, and plotted on a virtual grid of square pixels (10 × 10 m) [19]. The map was validated by ground-truthing a sample of patches within the study area and during the study period (see [19] for more details on the map creation). Given that we cannot measure variation in resource production in real time in every habitat type (because of dynamic grazing by many herbivore species and rainfall that may not be evenly distributed), we focused on how the two most preferred habitat types of wildebeest (grazing lawns and seep zones, [19]) were differently used. These habitats are spatially independent (S1 Fig. for a display of the spatial relationships among these vegetation types in the study area). Availability of high-quality resources around each GPS location was measured by the proportion of grazing lawns and the proportion of seep zones within 250 m of an animal’s location (see “Residence time and frequency of visits” below).

Predation risk was represented using distance to woody cover, at different times of day. The main predator species of wildebeest in this area is lion, an ambush predator that uses cover to hide and attack its prey, especially during the night [12]. Distance to cover thus represents a good proxy for predation risk. We used distance to the closest water point to investigate the influence of water availability, which is possibly also associated with a gradient in predation risk. The analyses including proximity to water were restricted to the dry season (early and late) months (during the wet season, water was widely available and this variable was therefore irrelevant). Distance to water during the dry season was estimated from monthly field surveys of water sources and aerial photos. Monthly field surveys were conducted to account for the complete drying of some smaller water sources during the course of the dry season.

### Residence time and frequency of visits

Resting bouts, defined as periods involving movements less than 50 m between successive hourly GPS locations and indicating that the animals had been resting rather than actively foraging or travelling, were excluded from further analyses. We estimated residence time (RT) to identify intensively used areas [14]. It corresponds to the time spent in a virtual circle with a fixed radius sliding along the path. It is computed as the sum of the first passage duration (i.e. forward minus backward first passage times at the circle circumference) and the other (subsequent or previous) durations associated with the various portions of the path occurring within this circle, provided the time spent out the circle before the backward next passage time or after the forward previous passage time was less than a given threshold (see Fig. 1 in [14] for a conceptual diagram). A 250m radius was used in our analyses, corresponding to a circle area of approximately 20 ha. This radius was small enough to provide fine-scale resolution of habitat conditions and large enough to account for the herd as the sampling unit and avoid too much noise in the signals. A sensitivity analysis showed that our results are only marginally affected when using a radius half and twice the radius initially used (i.e. 125m and 500m in S4 and S5 Tables, respectively). We allowed excursions out of the circle before re-entering it for durations up to 6 hours (of active time) to include subsequent time spent within the circle in the RT. The choice of this time threshold is a tradeoff between noise and accuracy of the signal: the noise level decreases with the extra-time length, but this time also has to be sufficiently short to avoid counting different foraging events at close spatial locations but distant temporal location as a single event. As wildebeest usually drink once a day, a threshold of 6h active time is a sensible way to decrease noise while respecting natural space use patterns of wildebeest. When the tracked animal returned within the circle centred on a given location more than 6 hours after having left it, it was counted as a new visit (associated with its own residence time) to this location (see [15] for details). In this way, for each season, we computed the mean frequency of visits (FV) as the mean number of visits per month (total number of visits per season divided by the season duration expressed in month) to each 20 ha area surrounding any relocation.

Additionally, we measured the regularity of the visits as follows: if return visits occur randomly in time, the intervals between them should be distributed according to an exponential law with a mean equal to 1/FV and a median time equal to ln(2)/FV. In contrast, if visits are regularly spaced out in time or tend to be concentrated in bursts, the mean time should remain equal to 1/FV, but the median time should converge towards 1/FV in the former case and become smaller than ln(2)/FV in the latter case (note that the variance, which is classically used to distinguish between even, random and contagious distributions cannot be used here because the number of bursts can be very low (down to 1) and the variance obtained for a single burst can be as low as the one obtained for visits regularly distributed over the whole time interval). We therefore computed an index of Regularity of Visits (RV) in time as RV = FV*median time/ln(2), associated to every 20-ha areas surrounding a relocation. This index is equal to 1 for a random distribution, smaller than 1 when visits tend to be concentrated at some times in the season, and larger than 1 when visits tend to be regularly spaced out in time. It is worth noting that RT, FV and RV values are localized both in space and time, but by construction, FV and RV tend to show a high spatial consistency, and therefore are almost time independent: two relocations close in space (within 250 m) but distant in time can be attributed quite different RT values but will be attributed similar FV and RV values.

### Statistical analyses

The influences of environmental variables on RT, FV and RV were investigated using Linear Mixed Effect (LME) models. For all models, we also included the herd identity as a random effect to take into account the possible behavioural differences between herds. An autoregressive moving average structure (ARMA (1,1), [24]) was included in each model to account for the dependence of successive hourly relocations. We log-transformed RT and RV, and used the square root of FV, so as to obtain that each metric approximately follows a Gaussian distribution.

For each response variable (RT, FV and RV), we considered the effects of predictors related to functional consequences for resource acquisition, predator avoidance and surface water requirements (see Table 1). Given that we investigated the dynamic nature of movement processes, we included the interaction with season for all variables in all *a priori* models in addition to the simple effects for estimation of models parameters. For RT, we also included a three-class factor representing the time of day: “day” for daylight periods, “night” for darkness periods and “crepuscular” for dawn and dusk periods, during wet and dry seasons (S2 Fig.; note that because our purpose was to contrast day and night and for the sake of clarity on the figure, we only presented the predictions for day and night). We ran the models using data for all seasons first (“All seasons” in Table 1), then restricted data to the dry seasons to include distance to water (“Dry seasons” in Table 1). Two interaction factors that had ecological relevance in terms of predation risk were also considered: distance to wooded area × distance to water and distance to wooded area × time of day. Model selection procedures were applied first for each hypothesis independently (“Resources” and “Predation”) and the best models were then combined to build a “resource + predation” model (Table 1). The influence of each *a priori* model was then assessed (for all seasons first, then only for the dry seasons) using the Akaike Information Criterion approach [25], [26].

**Table 1:** Hypotheses tested and related variables used to build a priori models for foraging mechanisms of wildebeest in Kruger National Park.

	*Hypotheses*	*Variables description*	*Variables abbreviation*
*All seasons*	**[R]**	Season × proportion of grazing lawns	S × GL
		Season × proportion of seep zones	S × Seep
	**[P]**	Season × distance to wooded areas	S × Wood
		*Time of day × distance to wooded area*	*TD × Wood*
	**[R+P]**	Best model [R] + Best model [P]	
*Dry seasons*	**[RW]**	Season × proportion of grazing lawns	S×GL
		Season × proportion of seep zones	S×Seep
		Season × distance to nearest water point	S×Water
	**[PW]**	Season × distance to wooded areas	S×Wood
		*Time of day × distance to wooded areas*	*TD×Wood*
		Season × distance to water	S×Water
		Distance to wooded areas × distance to water	Wood×Water
	**[RW+PW]**	Best model [R] + Best model [P] + Best model [W]	
	**[RW+PW-W]**	Best model [R] + Best model [P] - variables related to water	

**[R]** = Resources hypothesis; number of *a priori* models: RT, FV and RV = 3. **[P]** = Predation hypothesis; number of *a priori* models: RT = 3, FV and RV = 1. [R+P] = “Resources + Predation” model. **[RW]** = “Resources and Water” hypothesis; number of *a priori* models: RT, FV and RV = 7. **[PW]** = “Predation and Water” hypothesis; number of *a priori* models: RT = 15, FV and RV = 7. **[RW+PW]** = “Resources and Water + Predation and Water” model. **[RW+PW-W]** = “Resources and Water + Predation and Water” model minus variables related to water. The set of models were tested for all seasons first on each movement metric (RT, FV and RV, see Material & Methods) and then on the two periods of the dry season (early and late) to include the possible influence of water. Variables in italic only had biological meaning for RT and were only included in the model selection procedures for this metric. Although we included interactions with season for all variables in the model selection procedure, simple effects of each variable were systematically included for estimation of model parameters.

Finally, we investigated the joint combination of RT and FV values (for each relocation) among seasons and among vegetation types by categorizing them in three classes. Locations with RT among the 40% highest values were labelled “HRT”, and those within the 40% of the lowest values were labelled “LRT”. The same procedure was applied to FV, to determine “HFV” and “LFV” relocations. Each relocation was then either assigned to one of 4 categories: HRT-HFV, HRT-LFV, LRT-HFV and LRT-LFV or not considered (intermediate values in RT and/or FV, so as to increase the contrast in subsequent analyses). For each herd independently, we then tested if the distribution of relocations in the four categories depended on the seasons and on the vegetation types using chi² statistics. To avoid biases due to autocorrelation in RT and FV values, we used a bootstrap procedure where, for each herd, we randomly sampled 500 relocations and ran the chi² tests 1000 times.

## Results

**Fig 1:**
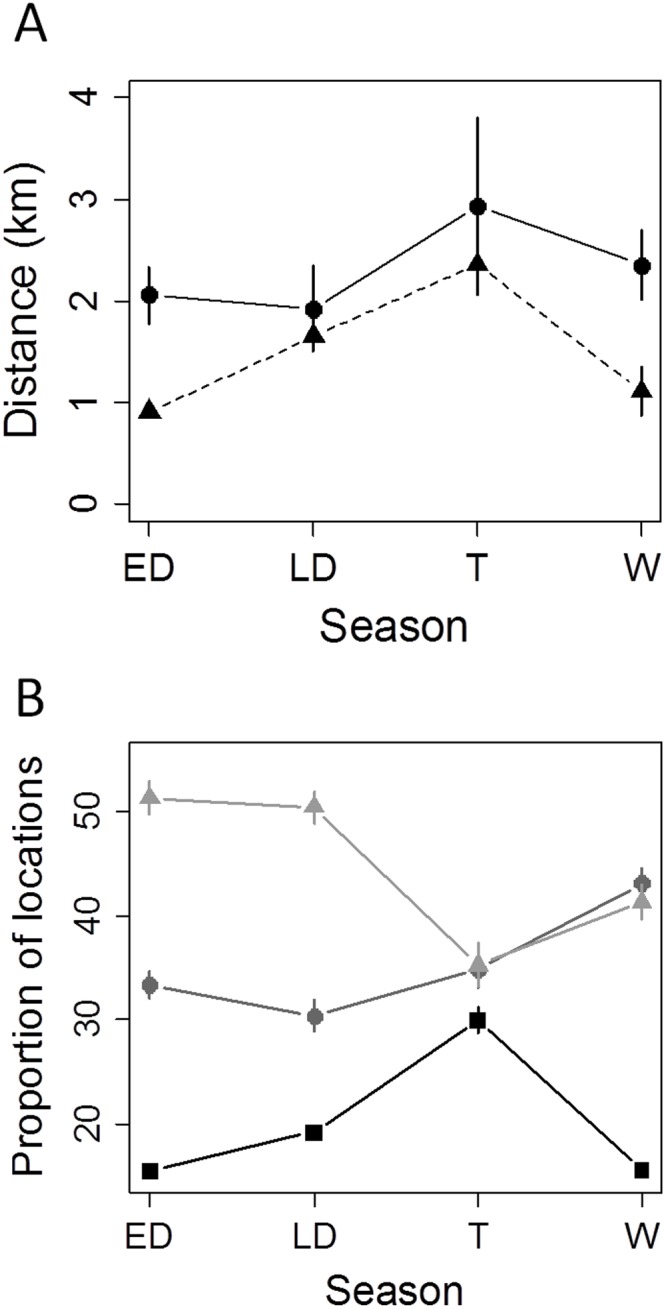
Daily distances travelled and daily net displacement (A) and behavioural budget (B) of wildebeest for each season. Mean ± SE (n = 9) are represented by black dots for daily distance travelled and black triangles for daily net displacement. On panel (B), mean ± SE (n = 9) of proportions of time involved in different behaviour are represented by gray triangles for resting, dark gray dots for foraging and black squares for travelling.

In average, wildebeests travelled slightly more per day during the wet season than during the late dry season, although their net displacement was lower (Fig. 1A). This trend was similar for the early dry season and wet season. The proportion of resting time decreased while the proportion of foraging time increased from early dry season to wet season. In contrast, the proportion of travelling was similar for early dry, late dry and wet seasons and much higher during the transition season (Fig. 1B). The ratio travelling/foraging was thus very different according to seasons: 0.47, 0.63, 0.86 and 0.36 for early dry, late dry, transition and wet seasons, respectively.

### Influence of environmental covariates on residence time (RT)

The median RT, estimated using active time only, were 6.4 ± 0.4 h, 4.2 ± 0.2 h, 3.7 ± 0.2 h and 7.9 ± 0.4 h (mean ± SE, n = 9) for early dry, late dry, transition and wet seasons, respectively.

**Fig 2:**
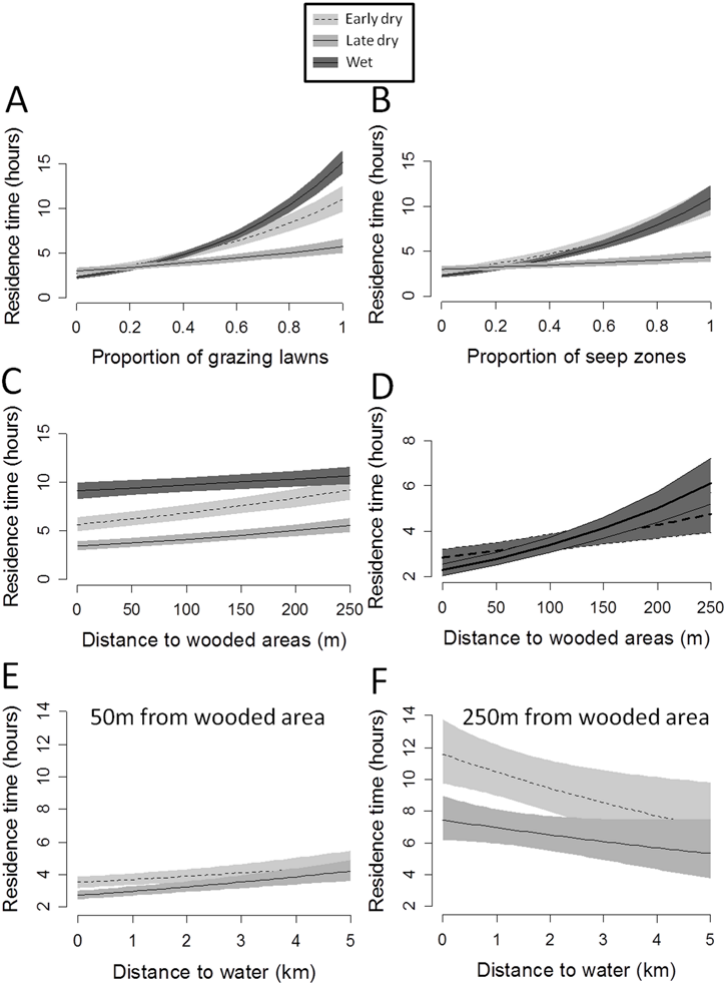
Predictions of linear mixed models of the influence of covariates on the residence time. (A) interaction season × proportion of grazing lawns, (B) interaction season × proportion of seep zones, (C) interaction season × distance to wooded areas, (D) interaction distance to wooded area × time of day, (E) and (F) interaction distance to wooded area × the nearest water point on the residence time in a circle of 250 m radius around GPS relocations of wildebeest in Kruger National Park.

For all seasons, the best model for RT was the model including factors representing both resources and predation risk (Table 2). The RT increased significantly with the proportion of grazing lawns in a 250 m radius circle around the location and was higher during wet and early dry seasons than late dry season (Fig. 2A). The RT also increased significantly with the proportion of seep zones, although less strongly than for grazing lawns (*c*.*a*. 3 active hours less for a circle composed of 80% seep zone compared to 80% of grazing lawns; Fig. 2B).

**Table 2:** Best models (with ΔAIC < 2) after model selection for residence time, frequency of visits and regularity of visits in a circle of 250 m radius around GPS relocations of wildebeest in Kruger National Park.

	*Hypotheses*	*Best models*	*AIC*	*ΔAIC*
**RT** *All Seasons*	**[R]**	S×GL + S×Seep	22859.1	193.8
	**[P]**	S×Wood + TD×Wood	23067.5	402.2
	**[R+P]**	S×GL + S×Seep + S×Wood + TD×Wood	22665.3	0
**RT** *Dry seasons*	**[RW]**	S×GL+ S×Seep + S×Water	12271.4	106.5
	**[PW]**	S×Wood + TD×Wood + S×Water + Wood ×Water	12164.0	53.1
	**[RW+PW]**	S×GL+ S×Seep + S×Wood + TD×Wood + S×Water + Wood×Water	12110.9	0
	**[RW+PW-W]**	S×GL+ S×Seep + S×Wood + TD×Wood	12646.5	535.6
**FV** *All Seasons*	**[R]**	S×GL + S×Seep	18455.1	132.5
	**[P]**	S×Wood	19370.0	1047.4
	**[R+P]**	S×GL + S×Seep + S×Wood	18322.6	0
**FV** *Dry seasons*	**[RW]**	S×GL+ S×Seep + S×Water	10269.9	106.8
	**[PW]**	Wood×Water	10488.4	325.3
	**[RW+PW]**	S×GL+ S×Seep + Wood×Water + S×Water	10163.2	0
	**[RW+PW-W]**	S×GL+ S×Seep + Wood×Water + S×Water	10779.2	616.0
**RV** *All Seasons*	**[R]**	S×GL + S×Seep	7880.4	66.2
	**[P]**	S×Wood	7934.2	120.0
	**[R+P]**	S×GL + S×Wood + S×Wood	7814.2	0
**RV** *Dry seasons*	**[RW]**	S×GL+ S×Seep + S×Water	3696.1	45.6
	**[PW]**	S×Wood + S×Water + Wood ×Water	3644.9	14.4
		S×Wood + Wood ×Water	3666.5	16.0
	**[RW+PW]**	S×GL+ S×Seep + S×Wood + S×Water + Wood×Water	3650.5	0
		S×GL+ S×Seep + S×Wood + Wood×Water	3652.3	1.8
	**[RW+PW-W]**	S×GL+ S×Seep + S×Wood	4115.8	465.3

**RT** = residence time; **FV** = Frequency of visits; **RV** = Regularity of visits. **[R]** = Resources hypothesis; **[P]** = Predation hypothesis; **[R+P]** = “Resources + Predation” hypothesis; **[R+W]** = “Resources + Water” hypothesis; **[P+W]** = “Predation + Water” hypothesis; **[R+P+W]** = “Resources + Predation + Water” hypothesis; **[R+P-W]** = “Resources + Predation + Water” model minus variables related to water. ΔAIC corresponds to difference in AIC between the lower AIC among the best models for each hypothesis (e.g. **[R+P]** for the residence time for all seasons) and the best models of the other hypotheses (**[R]** and **[P]** for the same example).

The RT was shorter nearby wooded areas and increased with increasing distances from these areas, especially during early and late dry seasons (Fig. 2C). This pattern was more pronounced during daylight time (Fig. 2D). The best model for the analyses during dry seasons corresponded to the “resource + predation risk + water” hypothesis and included the interaction factors “season × distance to the nearest water point” and “distance to nearest wooded area × distance to the nearest water point” (Table 2). The RT was lower close to water, especially during late dry season (Fig. 2D). When the water point was close to a wooded area (50m), the RT was more than twice lower than when located farther (250 m; Fig. 2E).

Considering all seasons, resources explained RT more than predation risk, as represented by distance to woody cover (lower ΔAIC with the best model, Table 2). However, considering dry season only, water and predation were more important in explaining wildebeest RT (ΔAIC lower when removing from the model variables related to water and then to predation, than those to resources; Table 2).

### Influence of environmental covariates on frequency of visits (FV)

**Fig 3:**
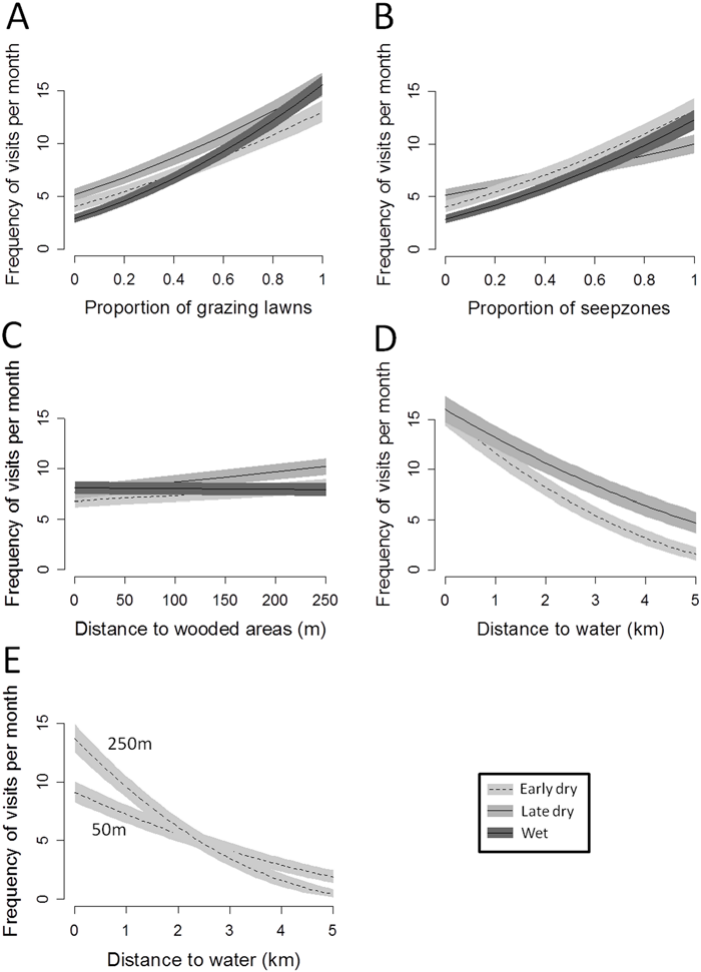
Predictions of linear mixed models of the influence of covariates on frequency of visits. (A) interaction season × proportion of grazing lawns, (B) interaction season × proportion of seep zones, (C interaction season × distance to the nearest water point, (D) interaction season × and distance to the nearest water point and (E) interaction distance to wooded area × distance to the nearest water point on the relative frequency of visits within circles of 250 m radius around GPS relocations of wildebeest in Kruger National Park. For panel (E), the labels “50 m” and “250 m” indicate the closest distance to wooded area.

For all seasons, the best model for FV was also the model including the “resource + predation risk” hypothesis (Table 2). The herds came back to the same areas more frequently when these areas encompassed higher proportions of grazing lawns, especially during wet season although the differences between seasons were not strongly marked (Fig. 3A). The same pattern was observed for the proportion of seep zones (Fig. 3B), but FV was less than for areas mostly composed of grazing lawns (*c*.*a*. 12 visits for 80% of grazing lawns in the circle *vs c*.*a*. 10 visits for 80% of seep zones). The wildebeests came back less often to places near wooded areas than places further, except during the wet season (Fig. 3C). The best model for the early and late dry seasons corresponded to the “resource + predation risk + water” hypothesis (Table 2). FV to locations close to water was equivalent during the early and late dry seasons. However, it was much lower for relocations that were also close to a wooded area (Figs. 3D and 3E). Whatever the season, resources and water explained FV more than predation risk (see ΔAIC Table 2).

### Influence of environmental covariates on regularity of visits (RV)

**Fig 4:**
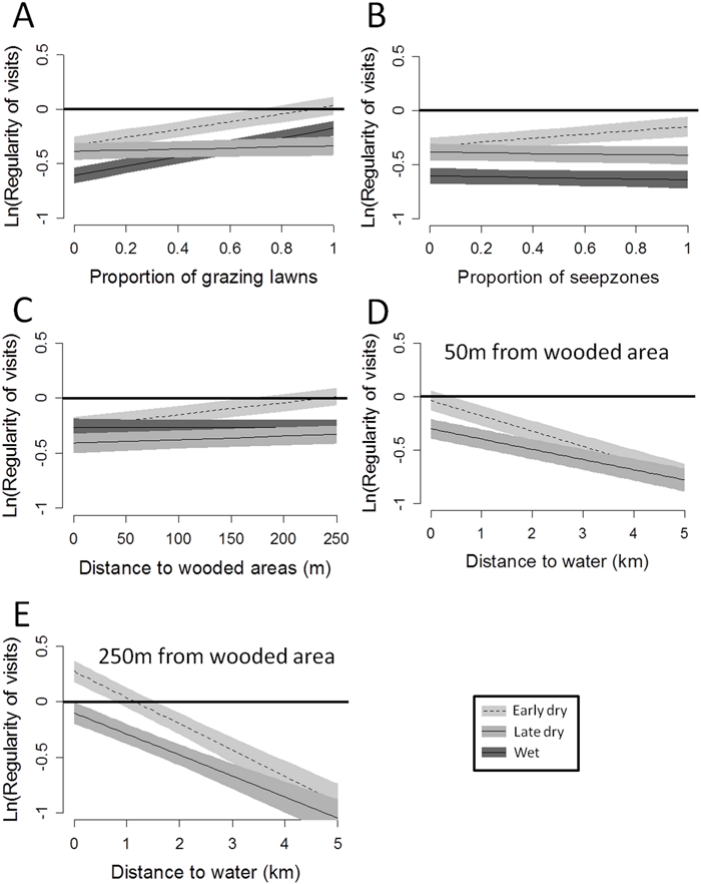
Predictions of linear mixed models of the influence of covariates on regularity of visits. (A) interaction season × proportion of grazing lawns, (B) interaction season × proportion of seep zones, (C) interaction season × distance to wooded area, (D) interaction season × distance to the nearest water point (50 m from wooded areas and (E) interaction season × distance to the nearest water point (250 m from wooded area) on regularity of visits within circles of 250 m radius around GPS relocations of wildebeest in Kruger National Park. To ease the interpretation of the figure, we kept the log-transformation of regularity of visits, meaning that random visits have an index close to 0, regular visits a positive index (maximum value: ln(1/ln(2) = 0.37 for extremely regular visits) and contagiously distributed visits a negative index (the more negative the index, the more the visits are aggregated).

For all seasons, the best model for RV corresponded to the “resource + predation risk” hypothesis (Table 2). The re-visits were aggregated in time when the proportion of grazing lawns in the circle was low but became more regular when this proportion increased (Fig. 4A). Visits tended to be more aggregated (i.e. less regular) during the wet season than during other seasons. Visits were also more aggregated but less influenced by the local proportion of seep zones during the wet and the late dry seasons, compared to the early dry season (Fig. 4B). The RV was not influenced by the distance to wooded areas during the wet and late dry season but strongly influenced during the early dry season, with more regular visits in areas farther from wooded areas (Fig. 4C). Wildebeest came back regularly to water points especially when the water points were located far from wooded areas and during the early dry season (Fig. 4D & 4E). Considering all seasons, without including water, resources explained the regularity of visits better than predation (Table 2). During the dry season, water was the main factor affecting the temporal structure of visits, followed by predation (see ΔAIC Table 2).

### Combination of residence time and frequency of visits

**Fig 5:**
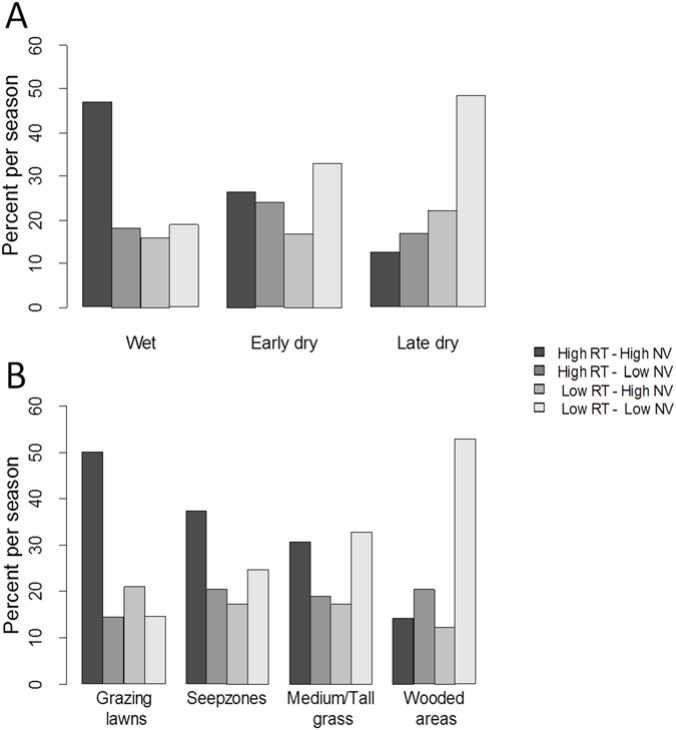
Distribution of classification of each GPS relocation based on their value for residence time and frequency of visits. Each class of GPS relocation is based on their value of residence time (RT) and frequency of visits (FV) within a circle of 250m radius around the relocation. (A) Distribution for the 3 seasons and (B) for the 4 vegetation types. The classification of each GPS relocation is based on 40% of the highest values (for high) or 40% of the lowest values (for low) of RT and FV. We weighted each class by the number of location per herd to account for the fact that some herds were followed over a longer period of time, in order to give the same weight for each herd.

For all herds, the distribution of relocations in terms of high or low RT and FV depended significantly on the season and on the vegetation type (S6 Table). During the wet season, a higher number of areas were often visited where herds also stayed for long (Fig. 5A). During the dry season, the proportion of relocations with high RT and high FV decreased and the proportion of locations with low RT and low FV increased, meaning that the herds did not stay long in a given area and seldom revisited these areas. Regarding vegetation types, we observed a decrease in proportion of relocations with high RT and high FV in seep zones compared to grazing lawns. This proportion was even lower for medium/tall grass and the lowest for wooded areas. Thus, wildebeests stayed foraging longer in grazing lawns and came back there more frequently than in seep zones. This pattern was even more pronounced in medium/tall grass. Wooded areas were mainly used for movement between patches of resources and/or water (Fig. 5B).

## Discussion

Factors representing food availability, exposure to predation and proximity to water all had an influence on the movements of the wildebeest herds. Wildebeest stayed longer in areas with higher proportions of both grazing lawns and seep zones, representing the key food resources in the wet season and late dry season, respectively [19]. However, contrary to prediction (a) (low residence times and high frequencies of visits during the wet season as compared to dry seasons), their residence time in these key resource areas was generally higher during the wet season than the dry season (especially the late dry season). The daily distance travelled was slightly higher in the wet season due to a lower proportion of resting time. As the proportion of travelling remained constant all year long (except during the short transition period), with respect to the dry season, the wet season is characterized by an increase in foraging at the expense of resting. The higher residence times in the wet season can thus be explained by the more frequent occurrence of foraging than in the dry season. Interestingly, the ratios median residence times (based on active time) over the proportion of active time (so as to take into account the increase of active time from dry to wet season) dramatically depended on the season: 13.1, 8.6, 5.7, and 13.4 for early dry, late dry, transition and wet season, respectively. This revealed less fragmented foraging sessions during the wet season compare to late dry and transition seasons, but similar foraging session during wet and early dry seasons.

This increase in the duration of foraging sessions may be the result of a higher nutritional demand associated with mating and calving but also lactating during this period [27], [28]. Indeed, the mating period occurs at the end of the wet season and the sharp birth peak 8–8.5 months later, i.e. at the beginning of the next wet season [27]. The movement constraints imposed by the calves during this period might also prevent the cows to move freely. However, wildebeest calves are followers and shortly after birth (about 7 min), they can stand and run [27]. Instead of the physical constraint that might constraints movement capacity, this behaviour could also reduce predation risk when food is not limiting.

Whatever the season, wildebeest also stayed longer in grazing lawns than they did in seep zones, partly contradicting prediction (b) (low residence times and high frequencies of visits in grazing lawns as compared to seep zones). Because the grass cover is typically taller in the seep zones, the grass is overall of lower nutritious quality. Wildebeest may therefore graze on the most nutritious grass within the seep zones but leave these areas quickly to keep foraging on high quality grass in other patches until the grass is not re-growing anywhere (end of early dry season and late dry season). However, as expected, the frequency of visits was much higher in grazing lawns than in seep zones and more regular in time during the wet and early dry seasons, suggesting that wildebeest were closely tracking grass re-growth in these areas. Recursion to previously visited areas is an effective foraging tactic to feed on re-growing grass [29].

These results partly contradict what we would expect from classical optimal foraging theory, and in particular the marginal value theorem, which predicts that in heterogeneous landscapes, animals should leave patches faster when the environment is globally richer and/or ranging time shorter [30]. Such predictions have been made to determine the optimal foraging strategy in a context where there are potentially an infinite number of patches, whose locations are not memorized, and are encountered at random. For selective grazers such as wildebeest that maximize their digestive rate [31] and restrict their movements within a home range, the number of potentially suitable patches is quite limited and their locations are certainly memorized. One can also hypothesize that wildebeest have probably some expectations about the quality of the various patches, but such expectations are probably partly unreliable because the patches are also exploited by other individuals of the same or different species. They should therefore tend to more or less regularly perform recursions to previously visited areas to track re-growing grass. In this context of pulsatile, thus less temporally predictable, production of high-quality resources, a more efficient tactic might be to stay longer in these patches than predicted by marginal value theorem, i.e. until they are almost entirely depleted. This rationale is especially relevant for species that closely track grass quality rather than biomass at fine-spatial scale, as shown for wildebeest in the Serengeti [31]. Surprisingly, wildebeest herds that continued to use grazing lawns during the dry season (5 out of 8 herds) re-visited these areas more frequently than during the wet season. This pattern highlights the stressful conditions encountered by wildebeest during this season, when they regularly visited areas of potentially good resources where they could not stay long because of food depletion and no grass production. Although they were less active during the dry season than during the wet season (50% *vs* 40% of the time spent resting), the daily distance travelled was only slightly lower than during the wet season (2 km *vs* 2.4 km for dry and wet seasons, respectively). Although during their active time in the late dry season, compared with the wet season, wildebeests moved more frequently between foraging sessions and spent less time foraging within the same area, they regularly re-visited areas previously exploited to track fine-scale changes in resource quality. This suggests that wildebeests tend to maximize their digestive rate instead of food ingestion rate, even during the stressful, dry season.

In addition to food quality and availability, predation is another factor that affects wildebeest movement. Wildebeests reduced their residence time near wooded areas especially during the night, as expected from the increased predation risk from ambush-predators such as lions under these conditions [12]. Anderson et al. [18] showed that elk residency rate was not correlated with preferred areas (with high forage biomass and far from predators), raising the hypothesis that it could be a counter-strategy to reduce predation risk by being less predictable. They suggested that if resources are not limited, animals should exhibit random residency rates to avoid predation whereas if resources are limited, residence time should increase in profitable areas [18]. We found the opposite pattern for wildebeests in this study, where they decrease their residence time during the dry season when resources are limited. Contrary to prediction (c) (predation as a main driver of movement during the wet season compared to dry seasons where food resources should be the main driver) proximity to water and predation avoidance explained the residence time and the visit regularity more than resources during the dry season when resources were limited. But as expected (d), distance to water was the main factor influencing wildebeest movements during the dry season. Wildebeest came frequently and more regularly near water but stayed less time when close to wooded areas and during late dry season when water points are scarce and can attract lions, as observed in Hwange [32]. They foraged further from water points between drinking events, presumably to avoid predation risk. This result is consistent with those from Valeix et al. [33] who found that the time spent accessing water for wildebeest in Hwange National Park (Zimbabwe) is shorter during the late dry season than during early dry season.

Suitable patches for herbivore species are usually tricky to define *a priori* unless using somehow subjective criteria. Animal movement has been widely used to identify profitable places and to define patches from the animal’s point of view (e.g. through identification of area-restricted search). In this study, we did not delineate profitable places for wildebeest but instead investigated the factors influencing the continuum of residence time and frequency and regularity of visits. So far, several movement metrics have been used to measure movements in relation to habitat, such as speed and turning angles, residence time (e.g. using an extended version of first passage time [14]), or path recursions (e.g. [13]), but always independently. A few studies have developed methods to decompose space use in terms of residence time and visit frequency [15], [34]. As far as we know, our present study is the first attempt to combine these two metrics to differentiate foraging tactics in response to environmental factors. This approach provides a new way of linking movement and habitat and highlighted the adaptive behavioural decisions made by wildebeest herds to exploit resources that are spatially and temporally heterogeneous and to avoid predation risk. It thus helps understanding how animals cope with changes in environmental conditions and may contribute towards identifying limitations on the number of individuals within the population. For example, in the Kruger National Park water hole management should account for the vegetation structure nearby as we showed that wildebeest movements around water points were influenced by proximity to woody cover, presumably as an anti-predator strategy during the dry season, a highly stressful period. These behavioural adaptations may affect individual fitness and therefore the population dynamics.

Our approach, when applied to other comparable populations (e.g. specialist grazers) thus should provide insights on the nature and consequences of the source of stress during winter/dry seasons. Our complementary analyses of different movement metrics provided an integrated view of changes in individual movement with varying environmental conditions and predation risk. Although we roughly estimated behavioural categories, it provided helpful insights to interpret movement responses. Further studies should include more accurate behavioural data (e.g. using 3D accelerometers) to link with these movement features, so as to fully identify the nature of and responses to environmental covariates. Understanding the complexity of these fine-scale spatial-processes allows mechanistic understanding of individual movements and the resulting population dynamics (emergence of large-scale patterns) and may thus contribute to conservation.

## Supporting Information

S1 Table Distance matrix between wildebeest herds in the Kruger National Park. These matrices report the distance between GPS relocations for the different wildebeest herds, showing their spatial independence. Each matrix reports the distances on the 1^st^ day of April, August and December during the study period (April 2009 to April 2011).
(DOC)

S2 Table Duration of GPS data recordings (gray cells) for each wildebeest herd in the Kruger National Park. n total correspond to the total number of GPS locations; n active correspond to the number of active locations and n circle to the locations (see text for more details). “n circle” corresponds to the locations for which the 250m circle did not overlap with the circles centred on locations within 6h before and after the current location (the time after which we consider a revisit in the circle; see text for more details).
(DOC)

S3 Table Classification of seasons. Classification of seasons during the GPS data recording of wildebeest herds in the Kruger National Park and rainfall (cm; mean ± SD).
(DOC)

S4 Table Model selection for circles of 125m radius. Best models (with ΔAIC < 2) after model selection for residence time, frequency of visits and regularity of visits in a circle of 125m radius around GPS relocations of wildebeest in Kruger National Park.
(DOC)

S5 Table Model selection for circles of 500m radius. Best models (with ΔAIC < 2) after model selection for residence time, frequency of visits and regularity of visits in a circle of 500 m radius around GPS relocations of wildebeest in Kruger National Park.
(DOC)

S6 Table Independence tests between combination of values of residence time and number of visits with season (column Season) and vegetation type (column Vegetation) for GPS location of wildebeest in the Kruger National Park. The *χ*² statistics result from bootstrapping procedures to avoid potential biases due to autocorrelation. For each herd, 500 values were randomly sampled 1000 times. The *χ*² statistics in the table is the mean *χ*² over the 1000 tests. *n* gives the number GPS locations from which the 500 values were sampled for each herd. For each test, the expected *χ*² for *α* = 0.05 is 12.6.
(DOC)

S1 Fig Principal component analysis computed on distances to the vegetation types of the study area. This analysis illustrates the spatial structure of the 4 vegetation types within the study area. The longest the arrows on an axis, the more the variables are correlated to this axis. The relative direction of the arrows on an axis indicates positive (same direction) or negative (opposite direction) correlation between two variables. Grazing lawns generally occur far from wooded areas, within matrix of medium and tall grass, whereas seep zones occur closer to wooded areas, and were spatially independent from grazing lawns.
(DOC)

S2 Fig Circadian activity pattern of wildebeest in the Kruger National Park. These patterns are based on distance travelled per active time (mean ± SE), during the wet (a) and (b) dry seasons. Red lines correspond to dawn and dusk. These patterns were used for the factor variable time of day: Day was set between 9h and 16h for wet season and between 10h and 16h for dry season; Night was set between 21h and 4h for wet season and between 5h and 20h for dry season.
(DOC)
