# Supplementary material for: Coping with Spatial Heterogeneity and Temporal Variability in Resources and Risks: Adaptive Movement Behaviour by a Large Grazing Herbivore
Source: PLoS One. 2015 Feb 26;10(2):e0118461. doi: 10.1371/journal.pone.0118461 (PMC4342283; doi:10.1371/journal.pone.0118461)
Supplement: S1 Table — These matrices report the distance between GPS relocations for the different wildebeest herds, showing their spatial independence. Each matrix reports the distances on the 1st day of April, August and December during the study period (April 2009 to April 2011). (DOC) [file pone.0118461.s001.doc]

**Supporting Information**

**S1 Table.** **Distance matrix between wildebeest herds in the Kruger National Park**. These matrices report the distance between GPS relocations for the different wildebeest herds, showing their spatial independence. Each matrix reports the distances on the 1^st^ day of April, August and December during the study period (April 2009 to April 2011).

| **April 2009** | **Herd 1** | **Herd 2** | **Herd 3** | **Herd 4** | **Herd 5** | **Herd 6** | **Herd 7** | **Herd 8** | **Herd 9** |
| --- | --- | --- | --- | --- | --- | --- | --- | --- | --- |
| **Herd 1** | - |  |  |  |  |  |  |  |  |
| **Herd 2** | 3539 | - |  |  |  |  |  |  |  |
| **Herd 3** | 3806 | 293 | - |  |  |  |  |  |  |
| **Herd 4** | 4131 | 4783 | 5035 | - |  |  |  |  |  |
| **Herd 5** | - | - | - | - | - |  |  |  |  |
| **Herd 6** | 962 | 4239 | 4482 | 4998 | - | - |  |  |  |
| **Herd 7** | - | - | - | - | - | - | - |  |  |
| **Herd 8** | - | - | - | - | - | - | - | - |  |
| **Herd 9** | - | - | - | - | - | - | - | - | - |

| **Aug 2009** | **Herd 1** | **Herd 2** | **Herd 3** | **Herd 4** | **Herd 5** | **Herd 6** | **Herd 7** | **Herd 8** | **Herd 9** |
| --- | --- | --- | --- | --- | --- | --- | --- | --- | --- |
| **Herd 1** | - |  |  |  |  |  |  |  |  |
| **Herd 2** | 13890 | - |  |  |  |  |  |  |  |
| **Herd 3** | 3289 | 11510 | - |  |  |  |  |  |  |
| **Herd 4** | 13906 | 43 | 11518 | - |  |  |  |  |  |
| **Herd 5** | - | - | - | - | - |  |  |  |  |
| **Herd 6** | 854 | 13827 | 2755 | 13840 | - | - |  |  |  |
| **Herd 7** | - | - | - | - | - | - | - |  |  |
| **Herd 8** | - | - | - | - | - | - | - | - |  |
| **Herd 9** | - | - | - | - | - | - | - | - | - |

| **Dec 2009** | **Herd 1** | **Herd 2** | **Herd 3** | **Herd 4** | **Herd 5** | **Herd 6** | **Herd 7** | **Herd 8** | **Herd 9** |
| --- | --- | --- | --- | --- | --- | --- | --- | --- | --- |
| **Herd 1** | - |  |  |  |  |  |  |  |  |
| **Herd 2** | 11077 | - |  |  |  |  |  |  |  |
| **Herd 3** | - | - | - |  |  |  |  |  |  |
| **Herd 4** | 6255 | 11650 | - | - |  |  |  |  |  |
| **Herd 5** | 6263 | 15112 | - | 4226 | - |  |  |  |  |
| **Herd 6** | 1022 | 11156 | - | 7266 | 7163 | - |  |  |  |
| **Herd 7** | 1736 | 12678 | - | 7395 | 6376 | 1578 | - |  |  |
| **Herd 8** | 10462 | 1200 | - | 11676 | 14904 | 10446 | 11998 | - |  |
| **Herd 9** | - | - | - | - | - | - | - | - | - |

| **Apr 2010** | **Herd 1** | **Herd 2** | **Herd 3** | **Herd 4** | **Herd 5** | **Herd 6** | **Herd 7** | **Herd 8** | **Herd 9** |
| --- | --- | --- | --- | --- | --- | --- | --- | --- | --- |
| **Herd 1** | - |  |  |  |  |  |  |  |  |
| **Herd 2** | - | - |  |  |  |  |  |  |  |
| **Herd 3** | - | 6824 | - |  |  |  |  |  |  |
| **Herd 4** | - | 9890 | 6092 | - |  |  |  |  |  |
| **Herd 5** | - | 13905 | 8998 | 4109 | - |  |  |  |  |
| **Herd 6** | - | 10072 | 3315 | 5639 | 6981 | - |  |  |  |
| **Herd 7** | - | 11163 | 4351 | 6486 | 7196 | 1180 | - |  |  |
| **Herd 8** | - | - | - | - | - | - | - | - |  |
| **Herd 9** | - | 2556 | 8610 | 12371 | 16333 | 11924 | 12932 | - | - |

| **Aug 10** | **Herd 1** | **Herd 2** | **Herd 3** | **Herd 4** | **Herd 5** | **Herd 6** | **Herd 7** | **Herd 8** | **Herd 9** |
| --- | --- | --- | --- | --- | --- | --- | --- | --- | --- |
| **Herd 1** | - |  |  |  |  |  |  |  |  |
| **Herd 2** | - | - |  |  |  |  |  |  |  |
| **Herd 3** | - | 6120 | - |  |  |  |  |  |  |
| **Herd 4** | - | 7868 | 5934 | - |  |  |  |  |  |
| **Herd 5** | - | 9697 | 5128 | 3623 | - |  |  |  |  |
| **Herd 6** | - | 9729 | 5156 | 3643 | 32 | - |  |  |  |
| **Herd 7** | - | 11986 | 5974 | 7902 | 4390 | 4380 | - |  |  |
| **Herd 8** | - | - | - | - | - | - | - | - |  |
| **Herd 9** | - | 4662 | 8147 | 12004 | 12939 | 12970 | 13929 | - | - |

| **Dec 10** | **Herd 1** | **Herd 2** | **Herd 3** | **Herd 4** | **Herd 5** | **Herd 6** | **Herd 7** | **Herd 8** | **Herd 9** |
| --- | --- | --- | --- | --- | --- | --- | --- | --- | --- |
| **Herd 1** | - |  |  |  |  |  |  |  |  |
| **Herd 2** | - | - |  |  |  |  |  |  |  |
| **Herd 3** | - | 5207 | - |  |  |  |  |  |  |
| **Herd 4** | - | 4090 | 6527 | - |  |  |  |  |  |
| **Herd 5** | - | - | - | - | - |  |  |  |  |
| **Herd 6** | - | 2836 | 5495 | 6894 | - | - |  |  |  |
| **Herd 7** | - | 4521 | 8568 | 8286 | - | 3118 | - |  |  |
| **Herd 8** | - | - | - | - | - | - | - | - |  |
| **Herd 9** | - | 11448 | 6267 | 11749 | - | 44534 | 14652 | - | - |

| **Apr 11** | **Herd 1** | **Herd 2** | **Herd 3** | **Herd 4** | **Herd 5** | **Herd 6** | **Herd 7** | **Herd 8** | **Herd 9** |
| --- | --- | --- | --- | --- | --- | --- | --- | --- | --- |
| **Herd 1** | - |  |  |  |  |  |  |  |  |
| **Herd 2** | - | - |  |  |  |  |  |  |  |
| **Herd 3** | - | - | - |  |  |  |  |  |  |
| **Herd 4** | - | - | 12141 | - |  |  |  |  |  |
| **Herd 5** | - | - | - | - | - |  |  |  |  |
| **Herd 6** | - | - | - | - | - | - |  |  |  |
| **Herd 7** | - | - | 3247 | 15246 | - | - | - |  |  |
| **Herd 8** | - | - | - | - | - | - | - | - |  |
| **Herd 9** | - | - | 115690 | 7625 | - | - | 14687 | - | - |
