# Supplementary material for: Coping with Spatial Heterogeneity and Temporal Variability in Resources and Risks: Adaptive Movement Behaviour by a Large Grazing Herbivore
Source: PLoS One. 2015 Feb 26;10(2):e0118461. doi: 10.1371/journal.pone.0118461 (PMC4342283; doi:10.1371/journal.pone.0118461)
Supplement: S2 Table — n total correspond to the total number of GPS locations; n active correspond to the number of active locations and n circle to the locations (see text for more details). “n circle” corresponds to the locations for which the 250m circle did not overlap with the circles centred on locations within 6h before and after the current location (the time after which we consider a revisit in the circle; see text for more details). (DOC) [file pone.0118461.s002.doc]

**Supporting Information**

**S2 Table**: **Duration of GPS data recordings (gray cells) for each wildebeest herd in the Kruger National Park**. n total correspond to the total number of GPS locations; n active correspond to the number of active locations and n circle to the locations (see text for more details). “n circle” corresponds to the locations for which the 250m circle did not overlap with the circles centred on locations within 6h before and after the current location (the time after which we consider a revisit in the circle; see text for more details).

|  | **2009** | | | | | | | | | **2010** | | | | | | | | | | | | **2011** | | | **n** | **n** | **n** |
| --- | --- | --- | --- | --- | --- | --- | --- | --- | --- | --- | --- | --- | --- | --- | --- | --- | --- | --- | --- | --- | --- | --- | --- | --- | --- | --- | --- |
|  | **04** | **05** | **06** | **07** | **08** | **09** | **10** | **11** | **12** | **01** | **02** | **03** | **04** | **05** | **06** | **07** | **08** | **09** | **10** | **11** | **12** | **01** | **02** | **03** | **total** | **active** | **circle** |
| **herd 1** |  |  |  |  |  |  |  |  |  |  |  |  |  |  |  |  |  |  |  |  |  |  |  |  | 8 132 | 3 706 | 556 |
| **herd 2** |  |  |  |  |  |  |  |  |  |  |  |  |  |  |  |  |  |  |  |  |  |  |  |  | 13 742 | 7 477 | 823 |
| **herd 3** |  |  |  |  |  |  |  |  |  |  |  |  |  |  |  |  |  |  |  |  |  |  |  |  | 13 460 | 6 849 | 1 055 |
| **herd 4** |  |  |  |  |  |  |  |  |  |  |  |  |  |  |  |  |  |  |  |  |  |  |  |  | 17 291 | 9 480 | 1 615 |
| **herd 5** |  |  |  |  |  |  |  |  |  |  |  |  |  |  |  |  |  |  |  |  |  |  |  |  | 9 236 | 5 593 | 798 |
| **herd 6** |  |  |  |  |  |  |  |  |  |  |  |  |  |  |  |  |  |  |  |  |  |  |  |  | 15 684 | 8 074 | 1 410 |
| **herd 7** |  |  |  |  |  |  |  |  |  |  |  |  |  |  |  |  |  |  |  |  |  |  |  |  | 13 174 | 7 713 | 1 238 |
| **herd 8** |  |  |  |  |  |  |  |  |  |  |  |  |  |  |  |  |  |  |  |  |  |  |  |  | 7 323 | 3 970 | 637 |
| **herd 9** |  |  |  |  |  |  |  |  |  |  |  |  |  |  |  |  |  |  |  |  |  |  |  |  | 7 506 | 4 120 | 183 |
