# Supplementary material for: Coping with Spatial Heterogeneity and Temporal Variability in Resources and Risks: Adaptive Movement Behaviour by a Large Grazing Herbivore
Source: PLoS One. 2015 Feb 26;10(2):e0118461. doi: 10.1371/journal.pone.0118461 (PMC4342283; doi:10.1371/journal.pone.0118461)
Supplement: S3 Table — Classification of seasons during the GPS data recording of wildebeest herds in the Kruger National Park and rainfall (cm; mean ± SD). (DOC) [file pone.0118461.s003.doc]

**Supporting Information**

**S3 Table: Classification of seasons.** Classification of seasons during the GPS data recording of wildebeest herds in the Kruger National Park and rainfall (cm; mean ± SD).

| **Season** | **Start date** | **End date** | **Rainfall** |
| --- | --- | --- | --- |
| Wet 1 | Start of GPS recordings | 24/04/2009 | 0.90 ± 3.7 |
| Early dry 1 | 24/04/2009 | 07/08/2009 | 0.39 ± 2.0 |
| Late dry 1 | 08/08/2009 | 26/10/2009 | 0.02 ± 0.1 |
| Transition 1 | 27/10/2009 | 26/11/2009 | 4.38 ± 11.2 |
| Wet 2 | 27/11/2009 | 06/05/2010 | 2.16 ± 6.5 |
| Early dry 2 | 07/05/2010 | 21/07/2010 | 0.10 ± 0.6 |
| Late dry 2 | 22/07/2010 | 06/11/2010 | 0.11 ± 0.5 |
| Transition 2 | 07/11/2010 | 06/12/2010 | 6.55 ± 13.2 |
| Wet 3 | 07/12/2010 | End of study | 2.77 ± 8.0 |
