# Supplementary material for: Coping with Spatial Heterogeneity and Temporal Variability in Resources and Risks: Adaptive Movement Behaviour by a Large Grazing Herbivore
Source: PLoS One. 2015 Feb 26;10(2):e0118461. doi: 10.1371/journal.pone.0118461 (PMC4342283; doi:10.1371/journal.pone.0118461)
Supplement: S4 Table — Best models (with ΔAIC < 2) after model selection for residence time, frequency of visits and regularity of visits in a circle of 125m radius around GPS relocations of wildebeest in Kruger National Park. (DOC) [file pone.0118461.s004.doc]

**Supporting Information**

**S4 Table**: **Model selection for circles of 125m radius**. Best models (with ΔAIC < 2) after model selection for residence time, frequency of visits and regularity of visits in a circle of 125m radius around GPS relocations of wildebeest in Kruger National Park.

|  | *Hypotheses* | *Best models* | *AIC* | *ΔAIC* |
| --- | --- | --- | --- | --- |
| **RT**  *All Seasons* | **[R]** | S×GL + S×Seep | 22 678.0 | 179.2 |
|  | **[P]** | S×Wood + TD×Wood | 22 899.1 | 400.3 |
|  | **[R+P]** | S×GL + S×Seep + S×Wood + TD×Wood | 22 498.8 | 0 |
| **RT**  *Dry Season* | **[R+W]** | S×GL+ S×Seep + S×Water | 12 098.2 | 166.6 |
|  | **[P+W]** | S×Wood + TD×Wood + S×Water + Wood ×Water | 12 046.8 | 115.2 |
|  | **[R+P+W]** | S×GL+ S×Seep + S×Wood + TD×Wood + S×Water + Wood×Water | 11 931.6 | 0 |
|  | **[R+P]** | S×GL+ S×Seep + S×Wood + TD×Wood | 12 464.3 | 532.7 |
| **FV**  *All Seasons* | **[R]** | S×GL + S×Seep | 18 292.9 | 118.9 |
|  | **[P]** | S×Wood | 19 203.1 | 1029.1 |
|  | **[R+P]** | S×GL + S×Seep + S×Wood | 18 174.0 | 0 |
| **FV**  *Dry Seasons* | **[R+W]** | S×GL+ S×Seep + S×Water | 10 110.2 | 2.1 |
|  | **[P+W]** | Wood×Water | 10 337.6 | 229.5 |
|  | **[R+P+W]** | S×GL+ S×Seep + Wood×Water + S×Water | 10 108.1 | 0 |
|  | **[R+P]** | S×GL+ S×Seep + Wood×Water + S×Water | 10 618.1 | 510.0 |
| **RV**  *All Seasons* | **[R]** | S×GL + S×Seep | 7 834.1 | 63.6 |
|  | **[P]** | S×Wood | 7 890.5 | 120.0 |
|  | **[R+P]** | S×GL + S×Wood + S×Wood | 7 770.5 | 0 |
| **RV**  *Dry Seasons* | **[R+W]** | S×GL+ S×Seep + S×Water | 3 665.7 | 43.1 |
|  | **[R+P+W]** | S×GL+ S×Seep + S×Wood + S×Water + Wood×Water | 3 637.1 | 14.5 |
|  |  | S×GL+ S×Seep + S×Wood + Wood×Water | 3 622.6 | 0 |
|  | **[R+P]** | S×GL+ S×Seep + S×Wood | 4 074.0 | 451.4 |

**RT** = residence time**; FV** = Frequency of visits**; RV** = Regularity of visits

[R] = Resources hypothesis; [P] = Predation hypothesis; [R+P] = “Resources + Predation” hypothesis; [R+W] = “Resources + Water” hypothesis; [P+W] = “Predation + Water” hypothesis; [R+P+W] = “Resources + Predation + Water” hypothesis. ΔAIC corresponds to difference in AIC between the lower AIC among the best models for each hypothesis (e.g. R+P for the residence time for all seasons) and the best models of the other hypotheses (R and P for the same example).
