# Supplementary material for: Coping with Spatial Heterogeneity and Temporal Variability in Resources and Risks: Adaptive Movement Behaviour by a Large Grazing Herbivore
Source: PLoS One. 2015 Feb 26;10(2):e0118461. doi: 10.1371/journal.pone.0118461 (PMC4342283; doi:10.1371/journal.pone.0118461)
Supplement: S5 Table — Best models (with ΔAIC < 2) after model selection for residence time, frequency of visits and regularity of visits in a circle of 500 m radius around GPS relocations of wildebeest in Kruger National Park. (DOC) [file pone.0118461.s005.doc]

**Supporting Information**

**S5 Table**: **Model selection for circles of 500m radius.** Best models (with ΔAIC < 2) after model selection for residence time, frequency of visits and regularity of visits in a circle of 500 m radius around GPS relocations of wildebeest in Kruger National Park.

|  | *Hypotheses* | *Best models* | *AIC* | *ΔAIC* |
| --- | --- | --- | --- | --- |
| **RT**  *All Seasons* | **[R]** | S×GL + S×Seep | 8 419.2 | 130.0 |
|  | **[P]** | S×Wood + TD×Wood | 8 532.8 | 243.6 |
|  | **[R+P]** | S×GL + S×Seep + S×Wood + TD×Wood | 8 289.2 | 0 |
| **RT**  *Dry season* | **[R+W]** | S×GL+ S×Seep + S×Water | 4 936.2 | 96.3 |
|  | **[P+W]** | Wood×Water | 4 889.5 | 49.6 |
|  |  | S×Wood + Wood ×Water | 4 890.5 | 50.6 |
|  |  | S×Water + Wood ×Water | 4 891.1 | 51.2 |
|  |  | S×Wood + Wood ×Water + TD×Wood | 4 839.9 | 51.4 |
|  | **[R+P+W]** | S×GL+ S×Seep + S×Water + Wood×Water | 4 839.9 | 0 |
|  | **[R+P]** | S×GL+ S×Seep | 5 141.3 | 301.4 |
| **FV**  *All Seasons* | **[R]** | S×GL + S×Seep | 6 010.7 | 90.3 |
|  | **[P]** | S×Wood | 6 256.9 | 336.5 |
|  | **[R+P]** | S×GL + S×Seep + S×Wood | 5 920.4 | 0 |
| **FV**  *Dry Season* | **[R+W]** | S×GL+ S×Seep + S×Water | 3 611.2 | 14.8 |
|  | **[P+W]** | Wood×Water | 3 687.8 | 91.4 |
|  |  | S×Wood + S×Water | 3 688.0 | 91.6 |
|  | **[R+P+W]** | S×GL+ S×Seep + Wood×Water + S×Water | 3 596.4 | 0 |
|  | **[R+P-W]** | S×GL+ S×Seep | 3 811.4 | 215.0 |
| **RV**  *All Seasons* | **[R]** | S×GL + S×Seep | 2 774.4 | 53.8 |
|  | **[P]** | S×Wood | 2 730.3 | 9.7 |
|  | **[R+P]** | S×GL + S×Seep + S×Wood | 2 720.6 | 0 |
| **RV**  *Dry Season* | **[R+W]** | S×Water | 1 368.5 | 0 |
|  |  | S×GL+ S×Seep + S×Water | 1 369.8 | 1.3 |
|  | **[P+W]** | S×Wood + S×Water + Wood ×Water | 1 334.4 | 34.1 |
|  | **[R+P+W]** | S×Wood + S×Water + Wood×Water | 1 368.5 | 0 |
|  | **[R+P-W]** | S×GL+ S×Seep + S×Wood | 1 496.8 | 128.3 |

**RT** = residence time**; FV** = Frequency of visits**; RV** = Regularity of visits

[R] = Resources hypothesis; [P] = Predation hypothesis; [R+P] = “Resources + Predation” hypothesis; [R+W] = “Resources + Water” hypothesis; [P+W] = “Predation + Water” hypothesis; [R+P+W] = “Resources + Predation + Water” hypothesis; [R+P-W] = “Resources + Predation + Water” model minus variables related to water. ΔAIC corresponds to difference in AIC between the lower AIC among the best models for each hypothesis (e.g. R+P for the residence time for all seasons) and the best models of the other hypotheses (R and P for the same example).
