# Supplementary material for: Coping with Spatial Heterogeneity and Temporal Variability in Resources and Risks: Adaptive Movement Behaviour by a Large Grazing Herbivore
Source: PLoS One. 2015 Feb 26;10(2):e0118461. doi: 10.1371/journal.pone.0118461 (PMC4342283; doi:10.1371/journal.pone.0118461)
Supplement: S6 Table — The χ² statistics result from bootstrapping procedures to avoid potential biases due to autocorrelation. For each herd, 500 values were randomly sampled 1000 times. The χ² statistics in the table is the mean χ² over the 1000 tests. n gives the number GPS locations from which the 500 values were sampled for each herd. For each test, the expected χ² for α = 0.05 is 12.6. (DOC) [file pone.0118461.s006.doc]

**Supporting Information**

**S6 Table**: **Independence tests between combination of values of residence time and number of visits with season (column Season) and vegetation type (column Vegetation) for GPS location of wildebeest in the Kruger National Park.** The *χ*² statistics result from bootstrapping procedures to avoid potential biases due to autocorrelation. For each herd, 500 values were randomly sampled 1000 times. The *χ*² statistics in the table is the mean *χ*² over the 1000 tests. *n* gives the number GPS locations from which the 500 values were sampled for each herd. For each test, the expected *χ*² for *α* = 0.05 is 12.6.

|  | *χ²* | | |
| --- | --- | --- | --- |
|  | *Season* | *Vegetation* | *n* |
| *Herd 1* | 103 | 141 | 1940 |
| *Herd 2* | 74 | 183 | 2470 |
| *Herd 3* | 109 | 136 | 3831 |
| *Herd 4* | 161 | 155 | 5247 |
| *Herd 5* | 67 | 136 | 2902 |
| *Herd 6* | 193 | 81 | 4662 |
| *Herd 7* | 113 | 271 | 4183 |
| *Herd 8* | 152 | 48 | 2150 |
| *Herd 9* | 77 | 137 | 957 |
