# Supplementary material for: Coping with Spatial Heterogeneity and Temporal Variability in Resources and Risks: Adaptive Movement Behaviour by a Large Grazing Herbivore
Source: PLoS One. 2015 Feb 26;10(2):e0118461. doi: 10.1371/journal.pone.0118461 (PMC4342283; doi:10.1371/journal.pone.0118461)
Supplement: S1 Fig — This analysis illustrates the spatial structure of the 4 vegetation types within the study area. The longest the arrows on an axis, the more the variables are correlated to this axis. The relative direction of the arrows on an axis indicates positive (same direction) or negative (opposite direction) correlation between two variables. Grazing lawns generally occur far from wooded areas, within matrix of medium and tall grass, whereas seep zones occur closer to wooded areas, and were spatially independent from grazing lawns. (DOC) [file pone.0118461.s007.doc]

**Supporting Information**

**S1 Figure: Principal component analysis computed on distances to the vegetation types of the study area**. This analysis illustrates the spatial structure of the 4 vegetation types within the study area. The longest the arrows on an axis, the more the variables are correlated to this axis. The relative direction of the arrows on an axis indicates positive (same direction) or negative (opposite direction) correlation between two variables. Grazing lawns generally occur far from wooded areas, within matrix of medium and tall grass, whereas seepzones occur closer to wooded areas, and were spatially independent from grazing lawns.
