# Supplementary material for: Coping with Spatial Heterogeneity and Temporal Variability in Resources and Risks: Adaptive Movement Behaviour by a Large Grazing Herbivore
Source: PLoS One. 2015 Feb 26;10(2):e0118461. doi: 10.1371/journal.pone.0118461 (PMC4342283; doi:10.1371/journal.pone.0118461)
Supplement: S2 Fig — These patterns are based on distance travelled per active time (mean ± SE), during the wet (a) and (b) dry seasons. Red lines correspond to dawn and dusk. These patterns were used for the factor variable time of day: Day was set between 9h and 16h for wet season and between 10h and 16h for dry season; Night was set between 21h and 4h for wet season and between 5h and 20h for dry season. (DOC) [file pone.0118461.s008.doc]

**Supporting Information**

**S2 Figure:** **Circadian activity pattern of wildebeest in the Kruger National Park**. These patterns are based on distance travelled per active time (mean ± SE), during the wet (a) and (b) dry seasons. Red lines correspond to dawn and dusk. These patterns were used for the factor variable time of day: Day was set between 9h and 16h for wet season and between 10h and 16h for dry season; Night was set between 21h and 4h for wet season and between 5h and 20h for dry season.
